# Protocol for a cross sectional study of cancer risk, environmental exposures and lifestyle behaviors in a diverse community sample: the Community of Mine study

**DOI:** 10.1186/s12889-019-6501-2

**Published:** 2019-02-13

**Authors:** Marta M. Jankowska, Dorothy D. Sears, Loki Natarajan, Elena Martinez, Cheryl A. M. Anderson, James F. Sallis, Stephen A. Matthews, Katie Crist, Lindsay Dillon, Eileen Johnson, Angelica Barrera-Ng, Kelsey Full, Suneeta Godbole, Jacqueline Kerr

**Affiliations:** 1 Calit2/Qualcomm Institute, UCSD, 9500 Gilman Dr, La Jolla, CA 92093 USA; 2 Nutrition, College of Health Solutions, Arizona State University, 445 N 5th Street, Phoenix, AZ 85004 USA; 3 Department of Family Medicine and Public Health, UCSD, 9500 Gilman Dr, La Jolla, CA 92093 USA; 4 UCSD Moores Cancer Center, 3855 Health Sciences Dr, La Jolla, CA 92093 USA; 5 Department of Sociology & Criminology, Department of Anthropology, Population Research Institute, Old Main, State College, PA 16801 USA

**Keywords:** Cancer, Built environment, Physical activity, Sleep, Diet, Insulin resistance, Inflammation, Global positioning system, Accelerometry

## Abstract

**Background:**

Physical inactivity and unhealthy diet are modifiable behaviors that lead to several cancers. Biologically, these behaviors are linked to cancer through obesity-related insulin resistance, inflammation, and oxidative stress. Individual strategies to change physical activity and diet are often short lived with limited effects. Interventions are expected to be more successful when guided by multi-level frameworks that include environmental components for supporting lifestyle changes. Understanding the role of environment in the pathways between behavior and cancer can help identify what environmental conditions are needed for individual behavioral change approaches to be successful, and better recognize how environments may be fueling underlying racial and ethnic cancer disparities.

**Methods:**

This cross-sectional study was designed to select participants (*n* = 602 adults, 40% Hispanic, in San Diego County) from a range of neighborhoods ensuring environmental variability in walkability and food access. Biomarkers measuring cancer risk were measured with fasting blood draw including insulin resistance (fasting plasma insulin and glucose levels), systemic inflammation (levels of CRP), and oxidative stress measured from urine samples. Objective physical activity, sedentary behavior, and sleep were measured by participants wearing a GT3X+ ActiGraph on the hip and wrist. Objective measures of locations were obtained through participants wearing a Qstarz Global Positioning System (GPS) device on the waist. Dietary measures were based on a 24-h food recall collected on two days (weekday and weekend). Environmental exposure will be calculated using static measures around the home and work, and dynamic measures of mobility derived from GPS traces. Associations of environment with physical activity, obesity, diet, and biomarkers will be measured using generalized estimating equation models.

**Discussion:**

Our study is the largest study of objectively measured physical activity, dietary behaviors, environmental context/exposure, and cancer-related biomarkers in a Hispanic population. It is the first to perform high quality measures of physical activity, sedentary behavior, sleep, diet and locations in which these behaviors occur in relation to cancer-associated biomarkers including insulin resistance, inflammation, impaired lipid metabolism, and oxidative stress. Results will add to the evidence-base of how behaviors and the built environment interact to influence biomarkers that increase cancer risk.

**Trial registration:**

ClinicalTrials.gov NCT02094170, 03/21/2014.

## Background

Physical inactivity, an unhealthy diet, and obesity are related to several cancers. In the United States, approximately 85,000 new cancer cases per year are related to obesity [1]. Research has found that as body mass index increased by 5 kg/m^2^, cancer mortality increased by 10% [2]. A healthy dietary pattern that is high in fiber and low in fat can reduce cancer risk [3, 4]. Physical activity (PA) has also been shown to be effective in the primary prevention of cancer, to prevent cancer recurrence, and to improve treatment effects [5, 6]. The largest review to date of the effects of PA on cancer outcomes found that individuals who participated in the most PA had 17% reduced risk for all cancer mortality in the general population and among cancer survivors [7]. More recently, sedentary behavior, independent of PA, has been related to cancer [8].

There are numerous biological mechanisms by which cancer is related to obesity and lifestyle factors. Obesity-related insulin resistance is associated with chronic systemic and tissue-localized inflammation and oxidative stress, which are thought to increase cancer risk [9]. Inflammation is known to promote the initiation and progression of cancer, and several insulin resistance and associated inflammation biomarkers have been shown to be biomarkers of cancer risk [10]. These include hyperinsulinemia [11], the HOMA-IR index [12], CRP, adipokines, and cytokines [13–15]. PA, sedentary behavior, diet and obesity are key, modifiable factors that influence insulin resistance, inflammation, and other cancer related biomarkers [16]. As such, understanding these modifiable lifestyle behaviors and their relationship to cancer is critical for minimizing incidence of obesity-associated cancers in high risk populations.

A 1998 review of the impact of PA interventions on population levels of behavior found that the majority of interventions focused on individual behavior change strategies had short term and limited effects on PA [17]. Reviews of individually-focused weight loss interventions also indicate that long term weight loss maintenance remains a challenge [18]. The Ecological Model of Behavior Change, which posits that individual health behaviors and outcomes exist within multiple levels of influence (individual, family, community, and policy), offers a lens through which to understand modifiable lifestyle behaviors beyond the individual [19, 20]. Research and worldwide health organizations (including WHO, CDC, IOM) have recommended an ecological approach to PA and healthy diet promotion and obesity prevention, including system level changes in policy and built environments [21]. Disparities in cancer mortality have also been considered from an ecological perspective including patient-, provider-, and health system-level factors [22, 23]. In Latino populations, research shows that PA levels are lower in racially segregated neighborhoods [24], and several studies have linked residential segregation and neighborhood-level SES to obesity [25, 26]. Understanding the role of environments in biological pathways leading to cancer can help identify environmental supports that facilitate the success of individual behavioral change approaches, identify locations where built environment changes are needed before individual efforts are implemented, and better recognize how environments may be fueling underlying ethnic and racial cancer disparities [27].

Research that considers built and social environmental factors like walkability, crime, parks, greenspace, and food environments in relation to lifestyle behaviors like PA and diet have presented mixed results [28–32]. Overall, the strongest relation has been found between walkability and walking for transportation [33]. Results have been less consistent for other environmental features such as parks and other behaviors such as total PA, with findings varying by population age, income and race [25, 29, 34]. Studies that relate walkability to obesity or BMI are inconsistent, especially in minority groups [35]. Food environment studies have focused mostly on access to foods in the neighborhood and demonstrate income inequalities in access with inconsistent associations with behaviors [36, 37]. Only two studies have assessed built environment and cancer risk factors such as insulin and diabetes [38, 39], with findings indicating that PA supportive environments are associated with better insulin and diabetes outcomes. The inconsistent findings to date may be due to: 1) poorly powered studies, 2) samples that lack exposure variation, 3) studies in limited population groups, 4) measurement error in behavior and environment, 5) lack of specificity in the hypothesized relations, 6) a focus on the residential neighborhood only, and 7) no inclusion of time spent in locations [34].

**Fig. 1:**
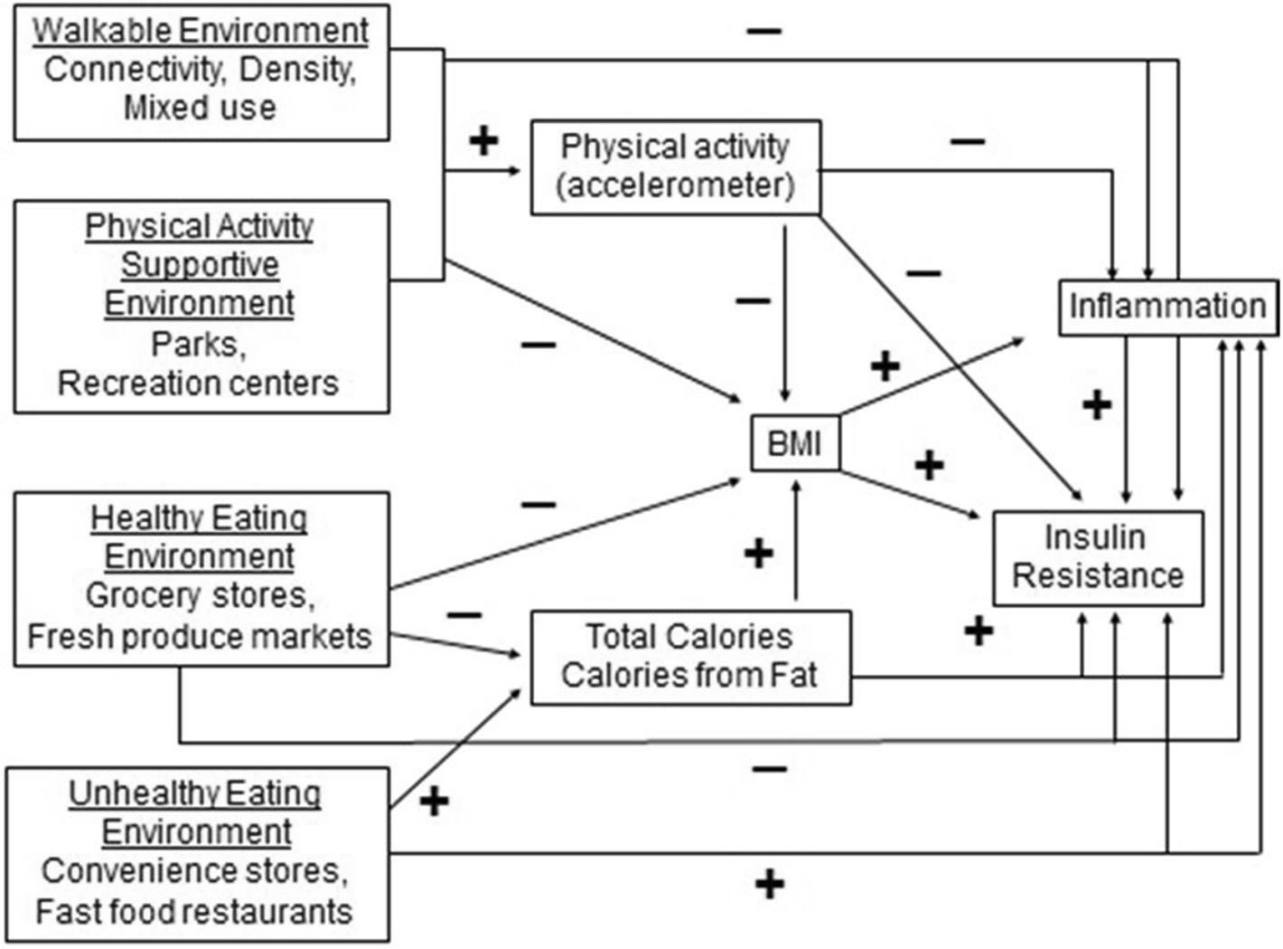
Hypothesized relationships between environments, behaviors, and biomarkers for the Community of Mine Study

This paper describes the protocol of a cross-sectional study of objective measures of PA, sleep, and environments, high quality measures of dietary intake, and biomarkers in Hispanic and non-Hispanic adults. The study completed recruitment in October of 2017, and is now in analysis phase. In total, 602 adults aged 35–80 years old were recruited, with 40% of the sample being Hispanic. The primary objective of the Community of Mine study is to advance methods of cancer risk exposure assessment by measuring both neighborhood access and total exposure to healthy environments by integrating Global Positioning System (GPS) data with Geographical Information System (GIS) data across the full day. These measures of exposure are expected to be associated with breast and colon cancer risk factors assessed through biomarkers, as well as PA and diet behaviors measured through accelerometry and food recalls. We hypothesize that dynamic minute-level GPS based measures of environmental exposures will be more strongly related to behavior, as well as insulin and inflammation biomarkers than static environmental measures based on participants’ home and work address. Figure 1 shows the hypothesized relationships between environments, behaviors, and biomarkers.

## Methodology

### Study design

This is a cross-sectional observational study conducted in San Diego County, California. Participants were randomly sampled from urban census block groups purposefully selected to maximize environmental variability in walkability and fast food access (as a proxy for healthy eating environments), and to strengthen our ability to uncover associations with cancer related outcomes. Walkability was derived from the San Diego Association of Governments’ (SANDAG) 2012 Health Atlas [40], which calculates a walkability index from net residential density, intersection density, retail floor area ratio, and land use mix. Versions of this index have been validated [41]. Data on food retail was obtained from San Diego County’s Department of Environmental Health’s Food Facility Inspection database in 2013. Terciles for walkability and fast food were calculated, and four categories were created using the highest and lowest terciles resulting in 764 census block groups for participant recruitment out of 1794 total in San Diego County: high fast food/low walkability (38 block groups), high fast food/high walkability (318), low fast food/low walkability (319), and low fast food/high walkability (89). No more than 10 participants per census block group were recruited. Recruitment began in 2014 and ended in 2017.

### Participants and eligibility

Participants include adults 35 to 80 years of age, of any ethnicity or race living for at least 6 months in a census block group selected for study. This middle aged and older adult population was selected because they likely have a more homogenous cancer risk profile (versus a younger group e.g. 18–40 years), but also provide increased exposure variability by including adults who are employed and likely to spend time outside of their home neighborhood. Adults over 80 years were not included as they are more likely to be home bound.

To be eligible, participants must be able to walk without human assistance, travel to a study visit, have a phone, be able to read and write fluently in English or Spanish, be able to give informed consent and comply with the protocol, and be willing and able to complete all assessments. Ineligible criteria are being pregnant or nursing, having a mental state that would preclude complete understanding of the protocol or compliance, having a medical condition that would affect PA or diet behavior, or having a medical condition known to increase inflammation biomarkers.

Recruitment for the study was conducted across the four walkability/fast food neighborhood types. Recruitment goals for each neighborhood type included 50% Hispanic, 50% female, and 50% in lower age range (35–60 years). Recruitment was initiated by obtaining name, age, gender, home address, and telephone number for individuals living within target census block groups from a marketing company. A letter was sent to potential participants informing them about the study and telling them to expect a phone call from study staff. Participants were contacted by experienced phone recruiters and given additional information on the study in either English or Spanish as appropriate. Participants who were interested and eligible were enrolled. All participants were provided with a copy of the study consent and HIPAA form and Subject’s Bill of Rights. These forms were reviewed section by section over the phone by trained research staff. Signed informed consent was obtained from all participants who enroll in the study.

### Study procedure

Once eligibility criteria were confirmed and the signed consent form was received, participants were scheduled for a clinical visit. A packet with devices, wear instructions, and instructions for the visit were delivered to participants approximately 1 week prior to the scheduled visit time. Participants were asked to wear an accelerometer on both the hip and wrist and a GPS device on a belt with the hip accelerometer for 14 days, with a minimum of 10 h of wear time per day. Writs accelerometers were requested to be worn at all times including during sleep. They were asked to complete a sleep log for each night. Participants were asked to fast for 12 h prior to the visit for a 45 mL blood draw and urine (~ 50 mL) sample. At the visit, blood pressure, height, weight, hip and waist circumference, temperature, and respiratory rate were recorded. Blood and urine samples were held on ice (EDTA tubes for 60 minutes, urine samples for 60 minutes, serum tubes for 30 minutes after 30 minutes at room temp to allow clotting) before centrifugation and aliquoting. Plasma, serum, and buffy coat from blood drawn into EDTA and serum tubes was isolated by centrifugation at 4 °C, then aliquoted and stored at − 80 °C. Urine samples were aliquoted and stored at − 80 °C.

During the clinical visit, participants completed a medical history interview that includes any current medications. Demographic characteristics (e.g., age, gender, race/ethnicity) were collected via self-report survey. Standard surveys measured health conditions, depressive symptoms, quality of life, sleep quality, fear of falling, and neighborhood perceptions. Participants received new devices at the clinic visit due to memory limitations on the devices. They were instructed to mail the devices back and, if at the end of the study they did not have at least 7 days of data, they were asked to re-wear the devices. Participants completed two 24-h dietary recall assessments (one weekday, one weekend). One dietary assessment was completed at the visit and the other was scheduled over the phone a few days after the visit. Participants received reminder calls to wear the devices and to allow them to ask questions. Once devices were returned participants were paid $100 for their participation. Participants also received $25 the day of their clinical visit to cover travel expenses and parking is provided.

### Biological outcomes

Insulin resistance and inflammation are the primary biomarker outcomes [10]. Insulin resistance is gauged by fasting plasma insulin and glucose levels, and the HOMA-IR index (fasting plasma insulin x fasting plasma glucose/22.5) [12]. Average 24 h circulating glucose levels were assessed by measures of HbA1c. Adipose tissue inflammation and insulin resistance are gauged by measuring the adipokines, adiponectin and leptin [42]. Systemic inflammation is gauged by levels of CRP (assayed in a multiplex panel with ICAM-1, VCAM1, and SAA – also markers of inflammation in insulin resistant subjects), [13, 43] IL-6, TNFα, and IL-10 (assayed in a multiplex panel with INFγ, IL-1β, IL-8, and IL-12p70 – also markers of inflammation in insulin resistant subjects), and MCP-1. IGF-1 axis is assessed measuring plasma IGF-1 and IGFBP - 3 levels. Impaired lipid metabolism is assessed by lipid panel measures of total, HDL and LDL cholesterol and triglycerides. Oxidative stress is assessed by urinary markers 8-oxo-dG and F2-isoprostanes [9, 44].

Anthropometric measures were taken in duplicate at the clinic visit. Height was measured without shoes using a stadiometer. Weight was measured without shoes using a bariatric digital scale, and waist circumference was measured along the midaxillary line using a flexible, non-stretchable measuring tape. Blood pressure was measured 3 times with 1 min between readings. A 4th measure was taken if two of three previous readings were > 5 mmHg different.

### Behavioral outcomes

Objective PA and sedentary behavior was measured by participants wearing a GT3X+ ActiGraph accelerometer (ActiGraph, LLC; Pensacola, FL) on the hip. Raw accelerometer data at 30 Hz is collected on 3 axes. Actigraph GT3X+ data are aggregated to the minute level using the low frequency extension. Participants wore the device for waking hours. During analysis, non-wear time is excluded using the validated Choi algorithm in Actilife 6 that clarifies invalid data as 90 consecutive minutes of zero counts with a 2 min tolerance and a 30 min small window to detect artificial movement [45]. The hip worn PA variables are created from collected accelerometer data employing traditional accelerometer count per minute (cpm) cut offs (e.g. moderate-vigorous PA 1952+; light intensity PA 100–1951+; and sedentary < 100) [46, 47], to be able to compare results to previous studies using these methods. However, we will also employ new machine learning techniques to classify specific behaviors related to built environments [48, 49]. These include walking/running, biking, sitting, standing still, standing moving, and sitting in vehicle. A random forest algorithm has been developed and tested by our group using a 10-fold cross validation on a training set with performance at 90% accuracy [50].

Objective sleep was measured by participants wearing a GT3X+ ActiGraph (ActiGraph, LLC; Pensacola, FL) on the non-dominant wrist for 24 h. Raw accelerometer data at 30 Hz is collected on 3 axes. Non-wear time is removed after being classified by 60 min of consecutive zeros on the vector magnitude [45]. Sleep duration and quality will be scored by visual inspection by trained raters, using the Actilife sleep analysis tab with the input of self-reported in-bed and out-of-bed times from participant sleep logs. After in-bed and out-of-bed time is determined by coder visual inspection, the Cole-Kripke algorithm [51] will be used to assess minute by minute sleep versus wake state. Variables include daily sleep duration, sleep onset time, sleep offset time, sleep efficiency, and minutes awake after sleep onset (WASO).

Objective measures of locations were obtained through participants wearing a Qstarz GPS device (BT-Q1000XT) attached to a belt worn on the waist. The device records data every 15 s. Missing data are imputed from a validated algorithm [52]. The GPS device logs geographic location coordinates, distance, speed, elevation, and time. The Qstarz has an industry reported accuracy of 3 m, and independent validation has shown that median error of the device varies by behavior (from 3.9 m for walking to 0.5 m for driving) and environment (from 5.2 m in urban canyons to 0.7 m in open areas) [53]. Participants were instructed to charge the GPS device overnight. The GPS data will be processed and joined to the accelerometer data using the Personal Activity and Location Measurement System (PALMS) [54]. Data will be aggregated and merged at the minute level. Indoor/outdoor locations will also be calculated using validated algorithms in PALMS [55]. The GPS device collects the signal to noise ratio of satellites in communication with the device. Higher ratios indicate more interference and likelihood of an indoor location. We have tested and validated a threshold of 250, which differentiates indoor and outdoor locations with over 85% accuracy compared to in person observations and coded images captures by person worn cameras.

Dietary measures were based on a 24-h multiple-pass food recall collected on 2 days by trained research staff using the NDSR (Nutrition Data System for Research) Software versions 2015 and 2016, a Windows-based program for food intake data entry and analysis developed by the Nutrition Coordinating Center at the University of Minnesota. The recalls were done on one weekday and one weekend day. Participants were given food portion visuals to aid in recall of portion sizes. NDSR allows research staff to collect a participant’s food intake for the previous day from midnight to midnight. The software guides the interviewer through its completion using a dynamic user interface. It is available in English and Spanish. A research staff member completed the master training course at the University of Minnesota on how to use the software and train others to administer the NDSR. Research staff participated in a rigorous training, and were assigned a set number of practices before completing the standardized test for certification. Ongoing monthly quality control checks of 10% of NDSRs were scheduled to maintain the quality of food interviews and data entry. Quality checks and staff feedback were conducted by the master trainer. All micro- and macronutrients are obtained, as well as data on foods and food groups. Reports per participant from NDSR will be used for primary analyses, and will include information on total calories, fat calories, fiber, macronutrients, and fruits and vegetables. Dietary outcomes from NDSR data include total calories, fat calories, fiber, and fruit and vegetable intake [56].

### Objective environmental exposures

The goal of the study is to better understand how environmental exposure to PA and diet-related environments impacts both behaviors and cancer-related biomarkers. Exposure in the study is measured in two ways: static measures around participants’ home and work locations, and dynamic measures using GPS traces of participants throughout the day. These two methods of measuring exposure will be compared to advance cancer exposure risk assessment. GIS data of the environment includes metrics of various land uses (retail, park, beaches, industrial, vacant), food outlet locations (grocery, fast food, restaurant, convenience store), walkability, recreation, public transportation, greenness, and crime. The data will be derived from the local government agency, SANDAG, as well as commercial data from Environmental Sciences Research Institute (ESRI) and Dun & Bradstreet.

Static measures of exposure are derived from buffers around participant homes as well as work places for those who work. The majority of studies that examine the relationship between environment and behaviors utilize a 400 m – 2 km buffer around a location to define an individual’s environment [34, 57]. A 1 km buffer around participants’ home and work locations will be created, and environmental variables contained in that buffer will be calculated for each participant. Static characterization of an individual’s environment does not accurately reflect a person’s daily mobility path and exposures that occur outside of the home and work environments, limiting the variance in environmental exposures over the study data collection period [58, 59]. Furthermore, single location estimates of exposure underestimate associations between environment and cancer risk factors when related to behaviors accumulated over the course of entire days, such as daily physical activity or total calories consumed [60, 61]. GPS devices offer a potential solution to these problems by providing a daily trace of an individual’s movement, which can be converted into a total dynamic exposure metric across the day. GPS-based dynamic measures of exposure will be calculated with the Kernel Density Equation (KDE) tool in ArcGIS. All GPS points for the entire participant’s wear time will be used to create a total exposure surface, which can then be multiplied by environmental variables to derive a total exposure measure that accounts for locations visited and the time spent in all locations [39].

### Self-report measures and covariates

Though the focus of this study is *objective* measurement, standard surveys items were administered for comparison with prior studies, because some perceptions e.g. safety and aesthetics are not available in objective GIS layers, and to assess important covariates. Surveys were provided in Spanish and English depending on language preference of the participant.

Demographic covariates include age, income, gender, ethnicity, education, employment status, employment location, marital status, number of children in the household, automobile ownership, driving status, type of residence, and years living at address. Family history of cancer was assessed with a standard population based surveillance survey [62], and employed as a covariate in analysis. Subjects were asked to record the names of all medications (prescription and over-the-counter) they currently take, and the doses of each (amount per day etc.). Participants were asked if a health professional ever diagnosed them with a chronic disease or risk factors. The Center for Epidemiologic Studies Depression Scale was included to assess symptoms of depression [63]. International Physical Activity Questionnaire *(*IPAQ *-* long form) assessed participants’ reported PA over the last 7 days. The IPAQ estimates PA frequency and duration per week for housework, occupation, transportation, and recreation [64]. The IPAQ includes a measure of total duration of weekday and weekend sitting time. Frequency and duration of sedentary behaviors were also be assessed by a separate 7-item measure [65]. Sleep disturbances was assessed using the NIH PROMIS Sleep Disturbance 6a Short Form and the MAP Index/ Survey Screen for Sleep Apnea. Quality of sleep was assessed using the Epworth Sleepiness Scale.

Due to the importance of the neighborhood design, an 11-item self-selection survey was deployed that measures reasons for moving to a neighborhood, which is a commonly cited potential bias in cross-sectional environmental studies [66]. Social interactions was measured using items from standard surveys assessing social isolation, cohesion, and support [67, 68]. Late Life Functioning Disability Instrument (LLFDI) was used to determine self-reported functional performance and physical disability [69]. The validated and widely used NEWS [70] was used to assess perceptions of neighborhood environment variables including subscales for aesthetics, and perceived safety from traffic and crime. The NEWS has good test-retest reliability (median ICC = .79) and construct validity by discriminating known neighborhood types and showing convergent relations to PA. Participants completed the NEWS scale for both home and work locations. For those who work, surveys were given related to support in the workplace for healthy food, physical activity and standing. The home sleep environment was assessed by the Functional Outcomes of Sleep Questionnaire (FOS-Q). Home sedentary environment included number of TVs in the bedroom etc.

### Sample size

Sample-size estimates were computed to achieve ≥80% power to detect meaningful differences in associations between outcomes and dynamic versus static GIS exposures. Specifically, a sample-size of 648 would achieve 80% power to detect a 0.32 correlation between outcome, Y, and Dynamic GIS-based neighborhood attributes (X) versus a 0.15 correlation between the outcome Y and Static GIS exposure (W). These calculations, based on the methods of Dunn and Clark [71] for dependent correlations, assumed a conservative correlation of 0.2 between the static and dynamic GIS-measures, and used a stringent significance level (α = 0.01 2-sided) to account for the multiple outcomes (BMI, MVPA, walking, and biomarkers). Further, accounting for census-block clustering effects with ICC = 0.05 to 0.1, we would require 630–660 participants. Recruitment was targeted at 700 individuals. Due to the intense data collection requirements, limited recruitment options due to environmental limitations (walkability and fast food access), and difficulty in recruiting Hispanic participants, recruitment fell short of the targeted 700 and the study was completed with 602 participants.

### Statistical approach

Our main objective is to compare Dynamic and Static GIS measures of PA and food environments and their associations with PA behaviors, BMI, diet, and biological markers. To test if the Dynamic GIS exposures are more strongly correlated with outcomes than the Static measures, we will apply the methods of Dunn and Clark [71] for dependent correlations (e.g., comparing two correlations which have a common variable, i.e., the PA behaviors or biomarkers). In addition, we will fit generalized estimating equations (GEE) models to examine associations between exposure to PA supportive environments (main effect) and outcomes (PA, BMI, diet, biomarkers) with adjustment for individual level confounding covariates (e.g. demographics, medication use for biomarker analyses), and block group clustering effects. We will use the multiple-informant approach developed by Horton et al. [72] to test if regression coefficients between PA and outcomes differ for the dynamic vs static GIS measures. We will examine interactions between covariates (e.g., ethnicity, age) and GIS measures on outcomes, to test if these factors moderate exposure-outcome associations. Similar analyses will be conducted for the self-reported outcomes, and Static versus Dynamic GIS measures of healthy eating promoting environments.

### Data storage and dissemination

Due to the sensitive nature of the GPS data collected, as well as the significant volume of data generated from the sensor devices, a secure geodatabase is used to store and process the GPS and accelerometer data. The geodatabase is housed in a HIPAA and FISMA-compliant (HIPAA Privacy Rule establishes national standards for protection individual medical records and personal health information) private computing cloud housed at the San Diego Supercomputer Center called Sherlock (http://sherlock.sdsc.edu). The geodatabase employs PostgreSQL to organize spatial data, and allow them to be directly exported to spatial software for visualization purposes [73]. All other data will be stored in a secure database, stripped of identifiers. Methods for data management and coding can be obtained by contacting the corresponding author. The results of the study will be disseminated through publications, reports, and conference presentations.

## Discussion

Understanding PA and dietary behaviors in the context of their environments can provide evidence leading to the design of potentially more effective multi-level interventions that can be designed to mitigate disparities in cancer outcomes. There are, however, still many limitations in the design and implementation of cross-sectional studies that examine PA and dietary behaviors in environmental contexts. There are no studies with extensive objective measures of behavior, environment, and exposure that also assess cancer-related biomarkers. The study is cross-sectional, limiting inferences of causality between outcomes. However, the environmental design and objective measurement of environmental exposures allows for more accurate assessment of associations between environment, behavior, and cancer-related biomarkers.

The Community of Mine study collected minute-level PA, sleep, and location data, food recalls, biomarkers, and survey information for 602 participants through a purposeful neighborhood-based sampling design, as well as extensive environmental information for the study area. The study protocol has completed data collection and is now moving into the data analysis phase of the study. It is important to note that there were significant challenges involved with data collection stemming from the large demands placed on participants (sensor device wear and clinical visits), limitations on location of recruitment (census block group walkability and fast food access selection design), and recruitment of Hispanic individuals. The location component of recruitment was purposeful to attempt to expand the variability in types of environments that participants lived in. However, with study results we hope to demonstrate that this may be an unnecessary recruitment limitation when the addition of GPS data may provide significant environmental exposure variability. While recruitment fell short of the 700 participant goal, the resulting data set is rich in objective measurements of health behaviors and biomarker outcomes. The main goal of the study is to advance methods of cancer risk exposure assessment by comparing the utility of static versus dynamic environmental exposures assessed by integrating GPS and GIS data. The data collected will allow for several other analyses of environmental exposure effects, such as time spent indoors and outdoors, interlinking effects of sleep duration/quality, PA, and diet, as well as environmental and racial inequalities as related to cancer risk.

## Abbreviations

CPM: Count per minute
ESRI: Environmental Sciences Research Institute
FOS-Q: Functional Outcomes of Sleep Questionnaire
GEE: Generalized estimating equations
GPS: Global positioning system
IPAQ: International physical activity questionnaire
KDE: Kernel Density Equation
LLFDI: Late Life Functioning Disability Instrument
NDSR: Nutrition data system research
PA: Physical Activity
PALMS: Personal Activity and Location Measurement System
SANDAG: San Diego Associations of Governments
